# Group hypnosis for stress reduction and improved stress coping: a multicenter randomized controlled trial

**DOI:** 10.1186/s12906-020-03129-6

**Published:** 2020-11-13

**Authors:** Silvia Fisch, Suzana Trivaković-Thiel, Stephanie Roll, Theresa Keller, Sylvia Binting, Margit Cree, Benno Brinkhaus, Michael Teut

**Affiliations:** 1 Psychotherapeutische Praxis, Daruper Str. 14, 48653 Coesfeld, Germany; 2 Institute for Social Medicine, Epidemiology, and Health Economics, Charité -Universitätsmedizin Berlin, Luisenstr. 57, 10117 Berlin, Germany; 3 MEDIAN Zentrum für Verhaltensmedizin Bad Pyrmont – Klinik für Psychosomatik, Bombergallee 10, 31812 Bad Pyrmont, Germany; 4 Institute of Biometry and Clinical Epidemiology, Charité-Universitätsmedizin Berlin, Charitéplatz 1, 10117 Berlin, Germany

**Keywords:** Hypnosis, Hypnotherapy, Stress reduction, Stress coping, Group program

## Abstract

**Background:**

The aim of the trial was to investigate the effect of a hypnotherapeutic group program in healthy persons with increased levels of perceived stress.

**Methods:**

In a randomized controlled multicenter trial participants with a self-assessed subjective stress level ≥ 40 mm on a visual analogue scale (0–100 mm; VAS) for the previous week and a stable state of health were randomized to either 5 weekly sessions of 120-min duration of a hypnotherapeutic group program for stress reduction and improved stress coping plus 5 hypnosis audiorecords for individual practice at home plus an educational booklet for stress coping (hypnosis group) versus an educational booklet only (control group). The primary outcome parameter was the VAS stress level for the previous week after 5 weeks. Secondary outcome parameters included the VAS stress level after 12 weeks, perceived stress (CPSS), depression (ADS-K), self efficacy (SWE) and quality of life (SF 36) after 5 weeks and 12 weeks. Analysis of covariance with a significance level of 5% using the full analysis set was used for analysis; the model included treatment (fixed effect), VAS baseline value (fixed covariate), and center (random effect).

**Results:**

A total of 95 participants were randomized; 47 (40 female, 45 ± 13.4 years of age) were allocated to the hypnosis group, and 48 (41 female, 46.9 ± 14.3 years) were allocated to the control group. Regarding VAS stress level after 5 weeks, the adjusted VAS mean in the hypnosis group was 41.8 mm [95% confidence interval (CI): 35.2; 48.4] compared to 62.9 mm [56.2; 69.7] in the control group, and the group difference was − 21.2 mm [− 30.1; − 12.2] (*P* < 0.001). After 12 weeks, the stress intensity on the VAS showed a between-group difference of − 14.7 mm [− 25.1; − 4.4] (*P* = 0.006), and the adjusted means were 41.1 mm [33.4; 48.8] in the hypnosis group and 55.9 mm [48.4; 63.5] in the control group. Improvements were also reported for CPSS, SF-36, SWE and ADS-K after 5 and 12 weeks.

**Conclusion:**

Compared to the control group, the hypnosis group showed reduced perceived stress after 5 and 12 weeks.

**Trial registration:**

ClinicalTrials.gov NCT03525093; date of registration: May 15, 2018.

## Background

Stress and stress-related diseases are considered important health issues worldwide [1–5]. A survey conducted in Europe showed that 20% of employees experience and perceive a high level of daily stress [3]. In Germany, a survey with 1200 adult respondents indicated that 61% of Germans reported being stressed often or sometimes, and 58% of the respondents reported recently feeling more stressed than they did 3 years before [4]. Chronic stress also plays a role in the development and aggravation of physical or mental illnesses. The German survey reported the following: “For respondents who described their state of health as less good or bad, almost one in three individuals often feels under stress. (...) Fifty-three percent of respondents who have had mental health problems in the past three years described themselves as ‘frequently stressed’” [4]. Popular stress management programs that have been tested for effectiveness are often based on a cognitive-behavioral approach [6–11]. An increasing number of stressed people are turning to complementary therapies, such as yoga, qigong, tai chi, meditation, and mindfulness-based stress reduction (MBSR). The effectiveness of these methods has been partially shown [12]. Additionally, hypnosis has become increasingly popular and has received greater worldwide attention in recent years. Research has shown evidence of the effectiveness of medical hypnosis in the context of several health conditions [13, 14]. The definition of hypnosis is “a state of consciousness involving focused attention and reduced peripheral awareness characterized by an enhanced capacity for response to suggestion” [15, 16]. Hypnotherapy is defined as “the use of hypnosis in the treatment of a medical or psychological disorder or concern” [15, 16] and currently includes a resource-activating and solution-oriented therapeutic attitude and a hypnosystemic style of speech [17, 18]. Despite many narratively reported positive experiences with using hypnotherapeutic interventions for stress reduction and improving stress-coping skills in the educational literature, there are only a few standardized hypnotherapeutic group programs available. Regarding its potential in stress reduction or prevention, there is currently a lack of high-quality clinical evidence. There are only a few studies that show positive effects of hypnotherapeutic interventions for stress reduction [16].

With the involvement of hypnosis experts, we designed a 5-session hypnotherapeutic group program for stress reduction and improved stress coping in healthy people with a perceived increased stress level in a prior feasibility study [5]. In the pre/post comparison of this exploratory observational study, a reduced stress level and an improvement in stress-coping competences were reported after 5 weeks. The results of this feasibility study led to the hypothesis that the designed hypnotherapeutic group program may reduce stress and improve stress-coping skills [5]. The aim of the present trial was to investigate the effectiveness of the hypnotherapeutic group program for stress reduction and improved stress coping in healthy persons with high levels of perceived stress.

## Methods

### Design

This study was a 2-armed randomized, controlled, open, multicenter trial performed at four study centers in Germany: 1. Hochschulambulanz für Naturheilkunde (Outpatient Department for Integrative Medicine) der Charité Universitätsmedizin Berlin; 2. a psychotherapeutic clinic in Coesfeld; 3. the Hospital of the Faculty of Medicine of the Westfälische Wilhelms-Universität, Münster; 4. MEDIAN Center for Behavioral Medicine - Department of Psychosomatics (Zentrum für Verhaltensmedizin – Klinik für Psychosomatik) in Bad Pyrmont. The study followed the guidelines for clinical trials and was approved by the ethics committee of the Charité Universitätsmedizin Berlin, Berlin, Germany (Approval No. EA1/067/18). The study was registered at ClinicalTrials.gov (Identifier NCT03525093). Participants provided written informed consent.

### Participants

Participants were eligible, if they fulfilled the following inclusion criteria: 18–70 years of age, self-assessed subjective stress level ≥ 40 mm on a visual analog scale (0–100 mm; VAS) for the previous week, a subjective increased level of perceived stress for at least 3 months, a stable state of health, and signed informed consent.

The following exclusion criteria were applied: current or planned participation in another psychological stress reduction program within the next 12 weeks, current use of psychotherapy, presence of moderate or severe acute or chronic disease conditions, and presence of an acute or chronic mental disorder.

Participants were recruited through newspaper advertisements in Berlin and Coesfeld, the website and newsletter of the Charité Outpatient Department for Integrative Medicine and the psychotherapeutic clinic in Coesfeld, the Newsletter of the Studienhospital Münster, and through flyers in the MEDIAN Zentrum Bad Pyrmont.

### Study interventions

After inclusion and baseline assessment, the participants in both groups received a written behavioral stress management educational booklet provided by a German health insurance company [19]. The information booklet (60 pages) contains the sections “recognizing stress”, “managing stress” and “preventing stress”. The section “recognizing stress” describes the physiological basis of a natural activation reaction and conveys various levels of a stress response (cognitive, emotional, vegetative, muscular). In addition, the reader is sensitized to the detection of individual stressors. The section “managing stress” introduces and briefly discusses common stress management strategies such as problem solving, time management, various relaxation techniques, sports, and recognizing and changing unfavorable attitudes. In the third section, “preventing stress,” the salutogenesis model is presented and the reader is informed about the structure and promotion of so-called resilience factors (in particular, the maintenance of social contacts). Finally, some short-term stress management strategies are explained, and a suggestion for a training protocol is given [5, 19].

The hypnotherapeutic group program was designed and tested in a prior feasibility study [5], and the concepts of the hypnotic trances contained ideas according to different authors [20–27]. It consisted of five standardized sessions covering health education, hypnotic inductions, and therapeutic talk (Table 1). The hypnotherapeutic group program was conducted by certified hypnotherapists (2 psychotherapists and 1 family physician) with groups between 8 and 12 participants in size in five weekly sessions of 120 min each. The hypnotherapeutic group program followed a manual with standardized hypnosis instructions that was developed and optimized in the prior feasibility study. In addition, prerecorded audio recordings (either a CD or downloadable MP3 files) of the hypnosis exercises were handed out to participants at the end of each session for self-practice at home. Participants were free to choose how and when to listen to the audio recordings (headphones, speakers, computers, CD players or other devices).

**Table 1:** Themes and procedure of the individual sessions of the hypnotherapeutic group program

Themes	Procedure	Duration (minutes)
**1. Session**		
Introduction to stress coping and experience of relaxation	• Getting acquainted, promotion of group cohesion and rapport	10
	• Psychoeducation on stress and stress management	35
	• Suggestibility test and convincer (“experiment with hypnosis”) a. Ideomotoric trance induction “magnetic hands” [20] b. Reorientation	15
	• Hypnosis: physical relaxation and mental reassurance a. Trance induction and deepening: mindful perception of body and posture, focus the attention on breathing [21] b. Distancing technique: balloon, in which one can let fly away one’s disturbing thoughts or themes c. Relaxation suggestions d. Mental reassurance: metaphor “mind as a pond” according to Stanton [22] e. Psychoeducation for the stress and relaxation reaction in a trance to promote the natural autonomic self-regulation f. Post-hypnotic suggestion to motivate participants to individual practice and homework g. Reorientation	30
	• Initiate self-employment of hypnosis recordings and farewell	30
**2. Session**		
Resource activation	• Exploration of the experiences with practicing independently at home	30
	• Imparting the principle of the “resource key for stress management”	10
	• Find necessary resources for the specific stressful situations of the participants	25
	• Hypnosis: dissociation from the stress situation and activation of a resource experience a. Trance induction and deepening: visual fixation, body scan [23, 24] b. Distancing technique: counting stairs to the resourceful place, box/suitcase to put off upsetting things or thoughts c. Resource identification and activation at the “resourceful place” d. Anchoring of the resource experience with a finger touch [23] e. Suggestion of helpful attitudes to stress management and achievement orientation according to Stanton [25] f. Post-hypnotic suggestion for repeated resource experience g. Reorientation	45
	• Completing the session and farewell	10
**3. Session**		
Resource key (Linking stressful situation and resource experience)	• Exploration of the experiences with practicing independently at home	30
	• Hypnosis: Repetition of the resource activation from the second session	40
	• Hypnosis: Practicing the rapid occurrence of resource experience	15
	• Hypnosis: Practicing the reflexive triggering of the resource experience through a stress stimulus (“resource key”) according to Bongartz’ “problem as anchor” [26]	25
	• Completing the session and farewell	10
**4. Session**		
Resource transfer (Experience of successful stress coping)	• Gathering and reinforcing the changing stress coping experiences of the participants	40
	• Hypnosis: Mentally anticipating and practicing a successful resource experience in a typical stress situation and coping with the stress situation (“resource transfer”) a. Trance induction and deepening, distancing technique: visual fixation; body scan; thoughts as clouds in the sky; noises, such as a radio in the background [23, 24]; stairs to the resourceful place b. Resource activation “resourceful place” c. Transfer of the resource experience into the critical situation: experience of successfully coping with the stress situation d. Post-hypnotic suggestion for successful coping with stress e. Reorientation	50
	• Completing the session and farewell	30
**5. Session**		
Future progression (Further improvement and stabilization)	• Appreciation of the changes in stress coping competences achieved so far and integration into self-image	45
	• Hypnosis: Facilitate further improvement and stabilization a. Trance induction with a marble [27] and deepening b. Partial age regression: Remembering the most important themes and experiences during the participation of the group program c. Age progression into a time in the future when the goal of improved coping with stress is reached: integration of competencies and characteristics in one’s self-image, increased self-efficacy d. Anchoring of this experience with a marble [27] e. Post-hypnotic suggestion for successful coping with stress f. Reorientation	35
	• Answer open questions and concerns	20
	• Completion and farewell	20

The intervention was intended to induce relaxation; to identify, activate and experience resources for coping with stressful situations; to develop and train stress-coping skills; and to apply mental training and anchoring [5].

The participants in the control group were offered free participation in the hypnotherapeutic group program after the study.

### Randomization

Patients were enrolled by the study physicians and study psychologists. After signing informed consent, inclusion in the trial, and baseline assessment, the participants were randomized to the intervention or control group in a 1:1 ratio via a central telephone randomization line by an otherwise independent study nurse. The randomization was stratified by center and in blocks of 20 participants (to take into account the group size of 10 people). The random allocation sequence was generated by using SAS 9.4 software (SAS Institute Inc. Cary, NC, USA).

### Outcome parameters

We used questionnaires with self reported outcome measures, which were filled out by patients at home and sent to the study office by post. The primary outcome parameter was the perceived stress level in the previous week on a visual analog scale (VAS; 0–100 mm: 0 no stress, 100 maximum stress) after 5 weeks [28, 29]. Construct validity can be assumed because of correlations between VAS and the subscales and total score of the Hospital Anxiety and Depression Scale HADS [30] of 0.66, 0.45 and 0.65, respectively [28, 29].

Secondary outcome parameters were VAS stress level after 12 weeks; Cohen’s Perceived Stress Scale (CPSS [31, 32];), a 10 items questionnaire that assess the degree to which people perceive their lives as stressful; score 0–40 with higher scores indicate a higher perceived stress level; depression assessed with the “Allgemeine Depressionsskala Kurzform “(ADS-K; score 0–45 with higher scores indicate higher level of depression) [33]; Schwarzer Self-Efficacy questionnaire (score 10–40, with higher scores indicate a better status of self-efficacy) [34]; and generic health related quality of life measured with the SF-36 questionnaire (score 0–100 with higher scores indicate better status of quality of life) [35, 36].

Furthermore, we asked participants about their most important individual goal they wanted to achieve with the hypnotherapeutic group program at baseline. We used Likert scales to assess the participants’ personal goal attainment and satisfaction with the intervention after 5 and 12 weeks. During the 12-week study period, the participants recorded the frequency of self-hypnosis exercises each week. After 5 weeks and after 12 weeks, participants were asked about the occurance of any critical life event during the last 5 and 7 weeks, respectively. Adverse events were recorded throughout the study observational period in the intervention and the control group.

### Statistics

Based on the previous feasibility study, we considered the difference in the primary endpoint (last week’s perceived stress intensity on a VAS) of 20 mm between intervention and control as a clinically relevant difference. With these assumptions, for a two-sided t-test with a significance level of 5%, a power of 90%, and an assumed standard deviation of 25 mm, a total of 34 participants per treatment group were necessary (68 in total). To compensate for an expected drop-out rate of approximately 15%, we planned to randomize 40 participants per group (80 in total).

Data analyses were carried out with the SAS for Windows 9.4 (SAS Institute, Cary, NC, USA). Statistical methods were defined in a detailed statistical analysis plan (SAP) before the data analysis. The analysis of the primary endpoint was performed by analysis of covariance (5% significance level), two-sided using the full analysis set (FAS) based on the intention-to-treat (ITT) principle without imputation of missing values; the analysis model included treatment (fixed effect), VAS baseline value (fixed covariate), and center (random effect). All further analyses were evaluated with similar models and were considered exploratory. A sensitivity analysis of the primary endpoint was performed with multiple imputations for missing primary outcome data.

## Results

### Participants

**Fig. 1:**
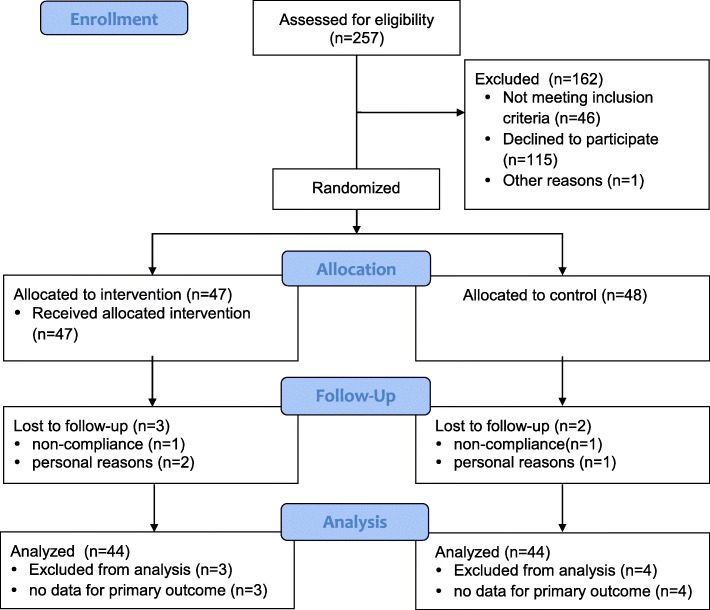
Participant flow diagram

The study was conducted between May and October 2018. Figure 1 shows the flow diagram of the participants. Of the 95 participants randomized, 47 were allocated to the hypnosis group and 48 to the control group. At the 12-week follow-up, 5 participants had dropped out of the trial (3 from the hypnosis group and 2 from the control group).

Table 2 shows the sociodemographic characteristics and Table 3 shows the baseline data of the outcome parameters for the participants.

**Table 2:** Baseline demographic characteristics (values are means ± standard deviations (SD) or absolute numbers (N) and percentages

	N	Hypnosis *N* = 47	Control *N* = 48	All participants *N* = 95
		Mean ± SD / N (%)	Mean ± SD / N (%)	Mean ± SD / N (%)
Age [years]	95	45.0 ± 13.4	46.9 ± 14.3	46.0 ± 13.8
Sex [female]	95	40 (85.1)	41 (85.4)	81 (85.3)
Education				
Abitur (German university entrance qualification)	95	36 (76.6)	37 (77.1)	73 (76.8)
Vocational education [university]	95	14 (29.8)	15 (31.3)	29 (30.5)
Employment				
Employed [yes]	95	41 (87.2)	39 (81.3)	80 (84.2)
Full-time employee	80	20 (48.8)	14 (35.9)	34 (42.5)
Part-time employee		21 (51.2)	25 (64.1)	46 (57.5)
Part-time because of stress [yes]	44	4 (20.0)	7 (29.2)	11 (25.0)
Incapacity for work last 4 weeks [no]	79	35 (87.5)	31 (79.5)	66 (83.5)
Size of household (more than single)	95	36 (76.6)	38 (79.2)	74 (77.9)
Health parameter				
BMI	95	23.3 ± 3.9	24.4 ± 4.2	23.8 ± 4.1
Smoking [yes]	95	7 (14.7)	9 (18.8)	16 (16.8)
Alcohol [yes]	95	37 (78.7)	39 (81.3)	76 (80.0)
Sports [yes]	95	44 (93.6)	46 (95.8)	90 (94.7)
Sport frequency [1–2 times weekly]	90	23 (52.3)	21 (45.7)	44 (48.9)
Stressful lifetime event in the last 6 months [yes]	95	21 (44.7)	19 (39.6)	40 (42.1)
Number of stress symptoms	95	8.7 ± 7.1 Range 3–39	8.4 ± 5.6 Range 3–25	8.6 ± 6.4 Range 3–39
Stress factors (multiple choices possible)				
Professional requirements				
Job/University	95	33 (70.2)	28 (58.3)	61 (64.2)
Exam preparation	95	10 (21.3)	8 (16.7)	18 (19.0)
High demands on oneself	95	35 (74.5)	33 (68.8)	68 (71.6)
Conflicts with colleagues / superiors	95	6 (12.8)	9 (18.8)	15 (15.8)
Time pressure, high density of appointments	95	32 (68.1)	16 (33.3)	48 (50.5)
Private requirements				
Private conflicts	95	18 (38.3)	17 (35.4)	35 (36.8)
Parenting	95	9 (19.2)	6 (12.5)	15 (15.8)
Disease (close people)	95	13 (27.7)	10 (20.8)	23 (24.2)
Care of a relative	95	5 (10.6)	3 (6.3)	8 (8.4)
Household	95	9 (19.2)	4 (8.3)	13 (13.7)
Money worries	95	5 (10.6)	7 (14.6)	12 (12.6)
Preparation special events	95	5 (10.6)	4 (8.3)	9 (9.5)
Adversities of everyday life/daily hassles				
Organization of everyday life	95	13 (27.7)	11 (22.9)	24 (25.3)
Public transport	95	7 (14.9)	8 (16.7)	15 (15.8)
Doctor visits	95	6 (12.8)	4 (8.3)	10 (10.5)
Waiting	95	1 (2.1)	6 (12.5)	7 (7.4)
Become disturbed / interrupted	95	16 (34.0)	7 (14.6)	23 (24.2)
Other	95	10 (21.3)	17 (35.4)	27 (28.4)

*N* numbers, *SD* standard deviation, *BMI* body mass index (kg/m^2^)

**Table 3:** Baseline characteristics (values are means ± standard deviations (SD))

	N	Hypnosis *N* = 47	Control *N* = 48	All participants *N* = 95
		Mean ± SD	Mean ± SD	Mean ± SD
VAS stress level [mm]^a^	95	73.8 ± 10.1	69.0 ± 11.3	71.4 ± 10.9
Perceived stress (CPSS score)^a^	95	22.5 ± 5.1	22.5 ± 5.9	22.5 ± 5.5
Depression (ADS-K score)^a^	95	26.9 ± 7.3	27.9 ± 6.9	27.4 ± 7.1
Self-Efficacy (SWE score)^b^	95	26.3 ± 5.3	25.2 ± 4.9	25.7 ± 5.1
SF-36^b^				
Physical Component Summary^b^	95	52.4 ± 10.0	50.7 ± 8.8	51.6 ± 9.4
Mental Component Summary ^b^	95	38.0 ± 9.7	38.4 ± 10.5	38.2 ± 10.1
SF-36^b^ Subscores				
Physical functioning	95	90.7 ± 14.7	91.3 ± 10.6	91.0 ± 12.7
Role limitations due to physical health	95	72.3 ± 31.8	71.4 ± 36.8	71.8 ± 34.3
Role limitations due to emotional problems	95	56.0 ± 36.2	61.8 ± 37.7	59.0 ± 36.9
Vitality	95	43.0 ± 17.0	43.1 ± 19.5	43.0 ± 18.2
Emotional well-being	95	56.9 ± 14.8	57.5 ± 15.1	57.2 ± 14.9
Social functioning	95	70.7 ± 23.9	63.0 ± 20.9	66.8 ± 22.7
Pain	95	76.3 ± 28.1	67.9 ± 27.1	72.1 ± 27.8
General health	95	61.2 ± 23.9	59.3 ± 20.6	60.2 ± 22.2

*VAS* visual analog scale, *CPSS* Cohen’s Perceived Stress Scale, *ADS-K* Allgemeine Depressions-Skala Kurzform (depression), *SWE* Selbstwirksamkeitserwartung (self-efficacy), *SF-36* Short-Form-Survey (quality of life)

^a^ lower values indicate better status, ^b^ higher values indicate better status

Most health-related parameters showed comparable values at baseline in both groups (Table 3).

### Primary outcome parameter

**Fig. 2:**
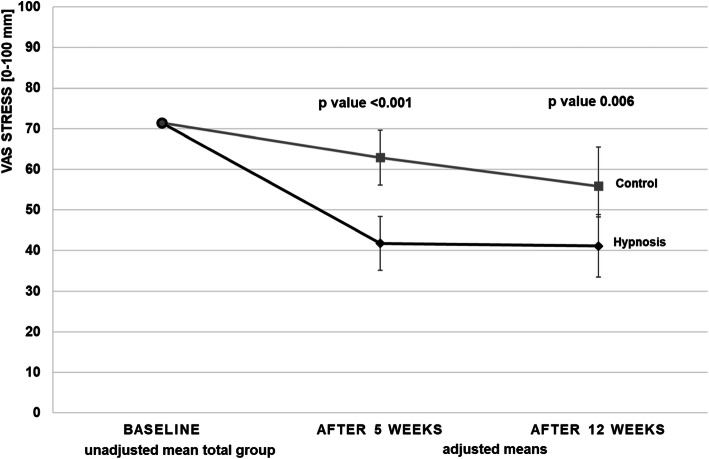
VAS = Visual Analogue Scale Stress (mm) at baseline (unadjusted mean of total group), after 5 weeks (primary outcome) and after 12 weeks (secondary outcome; both adjusted for respective baseline value and center) for hypnosis and control group

For the VAS stress intensity after 5 weeks (primary outcome parameter), there was a mean difference between the intervention and control group of − 21.2 mm [95% CI: − 30.1; − 12.2] (*P* < 0.001), with adjusted VAS means of 41.8 mm [35.2; 48.4] in the hypnosis group and 62.9 mm [56.2; 69.7] in the control group (Table 4, Fig. 2).

**Table 4:** Primary and secondary outcomes at week 5 for the hypnosis and control groups, means and mean group differences with 95% confidence interval (CI), adjusted for respective baseline value and center

		Hypnosis	Control	Control vs Hypnosis	
	N	Adjusted mean (95% CI)	Adjusted mean (95% CI)	Adjusted mean difference (95% CI)	*P* value
VAS stress intensity [mm]^a^	88	41.8 (35.2; 48.4)	62.9 (56.2; 69.7)	−21.2 (−30.1; -12.2)	<.001
Perceived stress (CPSS score)^a^	88	14.7 (13.1; 16.3)	20.3 (18.7; 22.0)	−5.7 (−7.8; −3.5)	<.001
Depression (ADS-K score)^a^	88	20.3 (18.5; 22.1)	25.1 (23.3; 26.9)	−4.9 (−7.2; −2.5)	0.001
Self-Efficacy (SWE score)^b^	88	30.00 (28.9; 31.1)	26.6 (25.5; 27.7)	3.4 (2.0; 4.9)	<.001
SF-36^b^					
Physical Component Summary^b^	88	51.6 (49.6; 53.6)	51.8 (49.8; 53.8)	−0.3 (−2.9; 2.4)	0.848
Mental Component Summary^b^	88	48.4 (45.7; 51.1)	39.2 (36.5; 41.9)	9.2 (5.6; 12.8)	<.001
SF-36^b^ Subscores					
Physical functioning	88	90.8 (87.9; 93.8)	91.1 (88.1; 94.1)	−0.3 (−4.2; 3.7)	0.897
Role limitations due to physical health	88	83.0 (73.8; 92.2)	72.8 (63.6; 82.1)	10.2 (−2.0; 22.4)	0.101
Role limitations due to emotional problems	88	80.1 (70.1; 90.0)	58.9 (48.8; 69.0)	21.2 (7.9; 34.6)	0.002
Vitality	88	60.1 (54.5; 65.6)	42.1 (36.4; 47.7)	18.0 (10.6; 25.4)	<.001
Emotional well-being	88	73.7 (69.4; 78.1)	59.7 (55.3; 64.1)	14.0 (8.3; 19.8)	<.001
Social functioning	88	82.7 (76.8; 88.7)	72.62 (66.7; 78.5)	10.1 (2.2; 18.1)	0.013
Pain	88	79.7 (73.3; 86.00)	74.2 (68.0; 80.3)	5.5 (−2.9; 13.9)	0.200
General health perception	88	68.7 (64.1; 73.4)	61.2 (56.5; 65.8)	7.6 (1.4; 13.7)	0.017

*CI* confidence interval, *VAS* visual analog scale, *CPSS* Cohen’s Perceived Stress Scale, *ADS-K* Allgemeine Depressions-Skala Kurzform (depression), *SWE* Selbstwirksamkeitserwartung (self-efficacy), *SF-36* Short-Form-Survey (quality of life)

^a^ lower values indicate better status, ^b^ higher values indicate better status

The additional sensitivity analysis of the primary outcome with multiple imputations for missing values showed adjusted means of 42.7 mm [36.0; 49.4] for the hypnosis group compared to 61.0 mm [54.3; 67.7] for the control group, and the mean difference was − 18.3 mm [− 27.3; − 9.3] (P < 0.001).

### Secondary outcome parameters

**Fig. 3:**
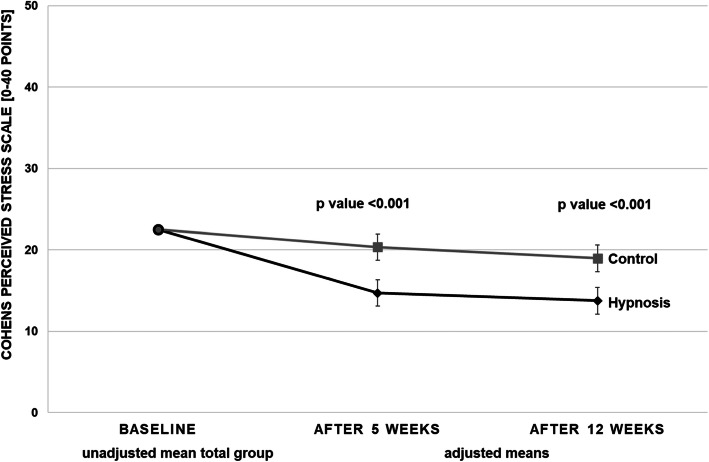
Cohen’s Perceived Stress Scale at baseline (unadjusted mean of total group) and after 5 weeks and 12 weeks (secondary outcome; both adjusted for respective baseline value and center) in the hypnosis and control groups

After 12 weeks, the stress intensity on the VAS showed a between-group difference of − 14.7 mm [− 25.1; − 4.4] (hypnosis group: 41.1 [33.5; 48.8]; control group: 55.9 [48.2; 63.5]; *P* = 0.006) (Table 5, Fig. 2). The hypnosis group also showed better values after 5 and 12 weeks for perceived stress (CPSS), depression (ADS-K), self-efficacy (SWE) and quality of life (SF-36 Mental Component Summary), but not on the SF-36 Physical Component Summary. The SF 36 subscores showed between-group differences for “Role limitations due to emotional problems”, “Emotional well-being”, “Vitality”, “Social functioning” and “General health perception” (Tables 4 and 5, Fig. 3). The evaluation of the frequencies of the participants who had increased levels of depression on the ADS-K (cut-off > 17 as a screening for depression, [33]) showed at baseline an increased ADS-K score in 93.6% of the hypnosis group patients and in 89.6% of the control group participants. After 5 weeks only 59.1% of the hypnosis patients had an ADS-K score > 17 compared to 86.4% of the control group patients (after 12 weeks 63.6% of the hypnosis group and 84.1% of the control group, respectively).

**Table 5:** Primary and secondary outcomes at week 12 for the hypnosis and control groups, means and mean group differences with 95% confidence interval (CI), adjusted for respective baseline value and center

		Hypnosis	Control	Control vs Hypnosis	
	N	Adjusted mean (95% CI)	Adjusted mean (95% CI)	Adjusted mean difference(95% CI)	*P* value
VAS stress intensity [mm]^a^	90	41.1 (33.5; 48.8)	55.9 (48.2; 63.5)	−14.7 (−25.1; −4.4)	0.006
Perception of stress (CPSS score)^a^	89	13.8 (12.1; 15.4)	19.0 (17.3; 20.6)	−5.2 (−7.4; −3.0)	<.001
Depression (ADS-K score)^a^	89	20.6 (18.9; 22.4)	25.4 (23.7; 27.1)	−4.8 (− 7.1; −2.5)	<.001
Self-Efficacy (SWE score)^b^	89	29.7 (28.6; 30.8)	26.4 (25.2; 27.5)	3.3 (1.8; 4.9)	<.001
SF-36^b^					
Physical Component Summary^b^	90	51.2 (48.9; 53.4)	51.4 (49.2; 53.5)	−0.2 (−3.1; 2.7)	0.895
Mental Component Summary^b^	90	49.3 (46.5; 52.2)	40.2 (37.4; 43.0)	9.1 (5.3; 12.8)	<.001
SF-36^b^ Subscores					
Physical functioning	90	90.4 (87.2; 93.7)	89.9 (86.8; 93.1)	0.5 (−3.8; 4.7)	0.829
Role limitations due to physical health	90	87.9 (79.7; 96.1)	73.9 (65.9; 81.9)	14.0 (3.3; 24.8)	0.011
Role limitations due to emotional problems	90	90.2 (80.5; 99.9)	67.2 (57.6; 76.8)	23.0 (10.2; 35.9)	0.001
Vitality	90	58.7 (52.8; 64.5)	44.4 (38.7; 50.2)	14.2 (6.5; 21.9)	0.001
Emotional well-being	90	74.6 (70.0; 79.2)	59.5 (55.0; 64.0)	15.1 (9.1; 21.1)	<.001
Social functioning	90	79.4 (72.4; 86.3)	70.0 (63.3; 76.7)	9.4 (0.2; 18.5)	0.046
Pain	90	78.9 (71.8; 86.1)	72.2 (65.4; 79.0)	6.7 (−2.7; 16.1)	0.160
General health perception	90	69.1 (64.6; 73.6)	63.8 (59.3; 68.2)	5.4 (−0.6; 11.3)	0.075

*CI* confidence interval, *VAS* visual analog scale, *CPSS* Cohen’s Perceived Stress Scale, *ADS-K* Allgemeine Depressions-Skala Kurzform (depression), *SWE* Selbstwirksamkeitserwartung (self-efficacy), *SF-36* Short-Form-Survey (quality of life)

^a^ lower values indicate better status, ^b^ higher values indicate better status

The participants in the hypnosis group stated high satisfaction with the program: after 5 weeks, 27.9% were satisfied, and 67.4% were very satisfied; after 12 weeks, 36.4% were satisfied, and 52.3% were very satisfied. The participants who completed the hypnotherapeutic group program participated in at least 4 sessions. Most of the participants practiced self-hypnosis with audiorecords 2–3 times per week during the 5-week intervention period. After completion of the group sessions, most participants continued their self-hypnosis practice up to the end of the trial at 12 weeks (36.4%, once a week; 52.3%, 2–3 times a week; and 11.4%, 4–5 times a week). After 5 weeks, 16 participants of the total sample (18.18%) reported the occurrence of a critical life event during the last 5 weeks (such as change of job, relocation, illness of a relative, loss of a person), after 12 weeks, 35 participants (38.89%) had a critical life event during the last 7 weeks. No adverse events were reported in this trial, that were associated with the intervention.

**Fig. 4:**
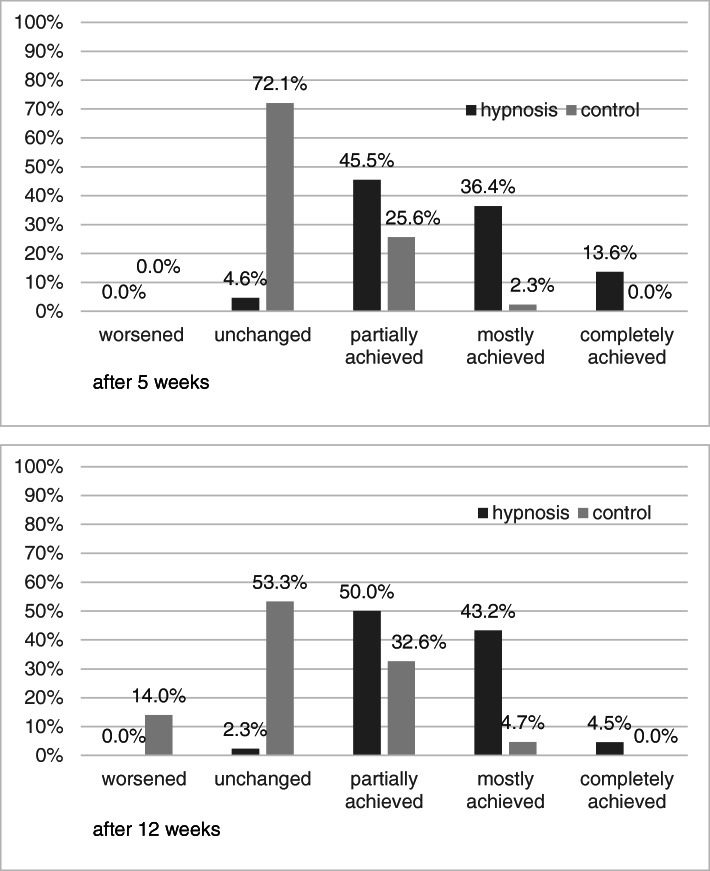
Personal goal attainment after 5 weeks. Percentages

After 5 weeks, 95.5% of the hypnosis group stated that their main therapeutic goal was partially or completely achieved (97.7% after 12 weeks) compared to 27.9% in the control group (37% after 12 weeks) (Fig. 4).

## Discussion

In this multicenter RCT, we observed statistically significant between-group differences in perceived stress intensity on a visual analog scale after 5 weeks and 12 weeks for participants in the hypnotherapeutic group program compared to individuals in the control group, who received only an educational booklet on stress management. In addition, we observed group differences favoring hypnosis in the secondary outcomes, such as perceived stress (CPSS), depression (ADS-K), self-efficacy (SWE), and quality of life (SF-36 Mental Component Summary), after 5 and 12 weeks. The results of the Likert scales for individual goal attainment and satisfaction with the hypnotherapeutic group program after 5 and 12 weeks fit well with the results. They showed that most participants were able to achieve their individual goals and were satisfied with the program. The participants showed good compliance and adherence (attendance and participation in the group sessions and individual practice of self-hypnosis with audio records during the 5 weeks of hypnotherapeutic group program duration and up to the end of the trial at 12 weeks).

While other studies on stress reduction with hypnosis have used interventions with more homogeneous groups (e.g., students, secretaries, high school teachers, or special groups of patients) [22, 25, 37–46] our hypnotherapeutic group program was designed for a wide range of healthy persons with high levels of perceived stress. The intervention was carefully developed by the study team on the basis of a systematic literature review [16] and the involvement of hypnosis experts with extensive experience as hypnotherapists and a broad knowledge of the professional literature on the topic. The hypnotherapeutic group program was conducted by qualified hypnotherapists (physicians or psychological psychotherapists). In comparison with other established stress management trainings using behavioral therapy with 8 to 12 group sessions of 2 h or 30 to 40 h in different course modules [7–9], our program is less time intensive. This might be of interest for healthy people, who already feel stressed and are looking for support to improve their stress management skills with manageable time requirements.

A limitation of this study is the impossibility of blinding the interventions, which could have introduced bias. The control group design, although providing a behavioral educational booklet for the control group, provided no control in regard to time, interpersonal contact, and expectancy. So it is impossible to assess which components of the intervention had a specific effect in reducing stress. Possible effect mechanisms of the intervention could have been the amount of time spent with the topic of stress reduction, the experience of group hypnosis, therapeutic expectations and suggestions, participating in a group with its group discussions and mutual support, received attention through the physician/psychotherapist, the hypnotherapeutic communication, and the individual self-hypnosis training at home. The low number of males participating in the hypnosis training and 85% female participation is particularly striking but consistent with the sex frequency distribution in our pilot study (86%, [5]) and with the experience in other studies with complementary medical interventions [16, 47]. It can be assumed that women may be more interested in hypnosis and more willing to participate than men. The education level, with 76.8% of participants completing German Abitur (high school), is also high. It is thus unclear whether the intervention would also be effective for a male population and for participants with a lower educational level.

Another weakness of the study is, that we did not assess the hypnotizability of participants in the hypnosis group by means of a suggestibility scale at baseline to compare the hypnotizability in both groups to examine a potential effect of hypnotizability as a moderator variable.

In contrast to other hypnosis studies on stress reduction [38–41], we did not assess laboratory data. This could be considered a weakness of our study, but we deliberately focused on patient-centered clinical outcomes. However, given the lack of blinding, physiological measurable stress variables would have been an additional tool for objectifying the assessment of stress. Furthermore the fact that the physician and psychotherapists who conducted the group programs also assessed the inclusion and exclusion criteria and health status at baseline, may have introduced some aspect of assessment bias.

A comparison of the data from the present study with the data of our sample from the feasibility study shows many similarities. In our former feasibility study, pre/post improvements in stress reduction (VAS stress intensity), perceived stress (CPSS score), depression (ADS-K) and quality of life (SF-36 Mental Component Summary) were reported. Additionally, in the SF-36 questionnaire, participants showed considerable improvements on the same subscores as in our current study, especially vitality and emotional well-being. The personal goal attainment measured by a Likert scale was similarly high in our previous study, in which 50% of the participants stated partially reaching their personal main goal and an additional 50% mostly or completely achieving their main goal. The satisfaction with the program was high as well (16.7% of the participants were satisfied and 83.3% were very satisfied) [5]. Thus, we could largely confirm the hypotheses derived from the exploratory results of our pilot study.

With a baseline ADS-K score of 27.4 ± 7.1, the overall sample showed a relatively high level of depression, which would be in line with the hypothesis that increased levels of subjective stress are often associated with depressive symptoms and can be considered a risk factor for developing a depressive disorder [48–52]. Also the frequency of participants that showed an increased level of depression with an ADS-K score > 17 at baseline was high with 93.6% of the hypnosis group and 89.6% of the control group, respectively. The fact that a difference in the degree of depression between the hypnosis group participants and the control group could be demonstrated after 5 weeks and 12 weeks, and that the frequency of ADS-K scores > 17 in the hypnosis group was reduced after 5 and 12 weeks compared to the control group allows the hypothesis that the hypnotherapeutic group program may be a useful tool for preventing the development of depressive symptoms.

In our study we found that the hypnosis program plus the behavioral stress management educational booklet was superior to the educational booklet alone in terms of reducing stress and improving stress coping. So the program could be useful if physicians and psychotherapists who have the appropriate hypnosis qualification could provide such a preventive stress reduction program in health education.

In German-speaking countries, a number of manualized, scientifically evaluated stress management trainings are available that are based on a cognitive-behavioral approach [6–11]. Usually, they combine psychoeducation modules, relaxation, time management, problem-solving training, enjoyment training, and cognitive restructuring. Overall, the research evidence has been positive for the effectiveness of these trainings [1]. A systematic review of 116 RCT studies, including multimodal training programs for the self-management of emotional stress, have demonstrated positive effects of cognitive behavioral stress management and cognitive behavioral therapy-based programs [12]. In addition, mindfulness-based stress reduction (MBSR) interventions [53, 54], are receiving increased attention. In meta-analyses, positive evidence has been found for stress reduction [55–57]. MBSR includes elements of instruction on mindfulness meditation, group dialogue aimed at enhancing awareness in everyday life and elements of mindful yoga.

Further studies should compare the effectiveness of this program with already established stress reduction programs (based on cognitive-behavioral therapy or MBSR) in non-inferiority trials. Additionally, basic research should explore the neurophysiological mechanisms of hypnosis in preventing and reducing stress. Furthermore, it would be interesting to assess more laboratory data as indicators for stress- and stress-related health factors (such as blood pressure and hormonal status). Additionally, the effectiveness in preventing a depressive disorder should be tested in further studies.

## Conclusion

In this multicenter randomized controlled trial, participants in a hypnotherapeutic group program for stress reduction and improved stress coping showed reduced perceived psychological stress measured by a visual analogue scale, Cohen’s Perceived Stress Scale, also reduction of depression scale and improved quality of life compared to individuals in a control group.

## Data Availability

The study protocol, the datasets used and/or analyzed during the current study are available from the corresponding author upon reasonable request. The hypnosis instructions used during the current study is already accepted for publication as a manual at Klett-Cotta, Imprint Schattauer publishers and will be published and available in 2021. The working title is “HypnoStressbewältigung – ein hypnotherapeutisches Gruppenprogramm”. The study was registered at ClinicalTrials.gov (NCT03525093) [58].

## Abbreviations

ADS-K: Allgemeine Depressionsskala Kurzform [General Depression Scale shortform]
CI: confidence interval
CPSS: Cohen’s Perceived Stress Scale
FAS: full analysis set
HADS: Hospital Anxiety and Depression Scale
ITT: intention-to-treat
MBSR: mindfulness-based stress reduction
N: numbers
p: probabilitas, probability
SAP: statistical analysis plan
SD: standard deviations
SF-36: Short-Form-Survey (quality of life)
SWE: Selbstwirksamkeitserwartung [self-efficacy]
RCT: randomized controlled trial
VAS: visual analogue scale
